# Sub-angiographic peripheral emboli in high resolution DWI after endovascular recanalization

**DOI:** 10.1007/s00415-020-09719-1

**Published:** 2020-01-29

**Authors:** Michael H. Schönfeld, Reza Kabiri, Helge C. Kniep, Lukas Meyer, Jan Sedlacik, Marielle Ernst, Gabriel Broocks, Tobias D. Faizy, Bastian Cheng, Götz Thomalla, Jens Fiehler, Uta Hanning

**Affiliations:** 1 Department of Diagnostic and Interventional Neuroradiology, University Medical Center Hamburg-Eppendorf, Martinistr. 52, 20246 Hamburg, Germany; 2 Department of Neurology, University Medical Center Hamburg-Eppendorf, Martinistr. 52, 20246 Hamburg, Germany

**Keywords:** Large vessel stroke, Mechanical thrombectomy, Embolism, Diffusion-weighted imaging

## Abstract

**Background and aim:**

To analyze the incidence of peripheral emboli after successful mechanical thrombectomy (MT) of intracranial large vessel occlusions (LVO).

**Methods:**

We performed a prospective analysis of patients with intracranial LVO who underwent successful MT and received a 1.5 T MRI including diffusion-weighted imaging (DWI) in standard- and high-resolution as well as susceptibility-weighted imaging (SWI) on the day following the intervention. Reperfusion grade was assessed on post-thrombectomy digital subtraction angiography (DSA) using the expanded thrombolysis in cerebral infarction (eTICI) scale. Punctuate DWI lesions distal to the DWI core lesion were classified as peripheral emboli. DWI lesions outside the primary affected vascular territory were classified as emboli into new territories. Additionally, SWI and post-thrombectomy DSA were analyzed and correlated to findings on DWI.

**Results:**

Twenty-eight patients undergoing successful MT met the inclusion criteria. In 26/28 patients (93%), a total of 324 embolic lesions were detected in DWI representing 2.1% of the cumulated ischemic core volume. 151 peripheral emboli were detected in standard-resolution DWI, 173 additional emboli were uncovered in high-resolution DWI. Eight out of nine patients with an eTICI 3 reperfusion had embolic lesions (29 DWI lesions). 9.6% (31/324) of peripheral emboli were observed in vascular territories not affected by the LVO. SWI lesions were observed in close proximity to 10.2% (33/324) of DWI lesions.

**Conclusions:**

Peripheral emboli are frequent after MT even after complete reperfusion. These emboli occur rather in the vascular territory of the occluded vessel than in other territories. A large proportion of peripheral emboli is only detected by high-resolution DWI.

**Electronic supplementary material:**

The online version of this article (10.1007/s00415-020-09719-1) contains supplementary material, which is available to authorized users.

## Introduction

Multiple randomized trials have demonstrated the benefit of mechanical thrombectomy (MT) for acute ischemic stroke due to large vessel occlusion (LVO) [1, 2]. Clinical outcome after MT can be improved by an increasing degree of reperfusion evaluated by the expanded thrombolysis in cerebral infarction (eTICI) scale [3, 4]. Accordingly, complete reperfusion (TICI 3) is leading to a markedly better outcome with the highest rate of excellent functional outcome (mRS ≤ 1) [5, 6].

Peripheral emboli as a complication after MT, supposedly caused by thrombus fragmentation, might worsen clinical outcomes [7]. In vitro data suggests that the majority of peripheral emboli released during MT are smaller fragments [8, 9]. Prevalence of distal emboli and infarcts in new territories are underestimated since most studies rely on computed tomography for follow-up [9]. Magnetic resonance imaging (MRI) in comparison is more sensitive in the detection of peripheral emboli that might not cause vessel obstruction detectable by digital subtraction angiography (DSA) [10]. An increase of spatial resolution of DWI termed high-resolution DWI has been shown to improve conspicuity and allows to detect more ischemic lesions than the standard-resolution DWI [11, 12]. This may give information of MT success beyond the eTICI scale. We aimed to analyze the incidence of peripheral emboli after successful reperfusion. We hypothesized that a substantial number of peripheral emboli lead to tissue ischemia within the vascular territory of an LVO and that these emboli can be detected on MRI more often than on DSA using high resolution diffusion-weighted imaging (DWI) and/or susceptibility-weighted imaging (SWI). This might be of importance for procedure-related safety features to prevent peripheral emboli during MT.

## Methods

The data that support the findings of this study are available from the corresponding author upon reasonable request.

### Study population

This was a prospective study of patients treated for intracranial LVO at our university hospital between 08/2017 and 11/2018. Inclusion criteria were defined as (1) MT for LVO within the anterior circulation; (2) successful reperfusion of eTICI ≥ 2b50; (3) postinterventional imaging including standard and high resolution DWI and SWI. The local ethics committee approved the study (Ethics Committee of the Physician Board Hamburg; approval number WF 019/19) and waived the requirement to obtain informed consent.

### Endovascular treatment

Some patients received IV alteplase prior to MT. Procedures were performed under general anesthesia or conscious sedation. The choice of thrombectomy devices, including balloon-guide catheter, were left to the operator’s decision.

### Magnetic resonance imaging (MRI)

MRI was performed on a 1.5 T MRI scanner (Siemens Magnetom Avanto, Erlangen, Germany) on the day following the intervention.

Axial DWI covering the whole brain was performed using single-shot, multi-slice, spin-echo, echo-planar imaging sequences with diffusion gradients in three orthogonal directions, b-values of 0, 500 and 1000 s/mm^2^, field of view 240 with the following alterations in protocols: a standard resolution DWI with a TR/TE 4800/84 ms, matrix 128 × 128, slice thickness 4 mm, no gap, with an acquisition time of 83 s and a high resolution DWI with a TR/TE 13,000/90 ms, matrix 192 × 192, slice thickness 2 mm, no gap, with an acquisition time of 457 s. Parameters of the high-resolution DWI were chosen to reach a voxel size comparable to other high-resolution DWI sequences [11, 13]. SWI was performed with a TR/TE 56/40 ms, matrix 260 × 320, thickness 2 mm, no gap, field of view 186.2 × 230 with an acquisition time of 325 s. SWI minimum intensity projections images were generated automatically by the scanner software.

### Imaging analysis

Post-interventional DSA imaging was rated with the expanded Thrombolysis in Cerebral Infarction (eTICI) definition as proposed by Liebeskind et al. with eTICI grade 2b50 defined as reperfusion of 50–66% of the downstream territory, grade 2b67 with 67–89% reperfusion, grade 2C with 90–99% reperfusion and grade 3 with complete or 100% reperfusion [3].

Peripheral emboli were defined as an area of signal drop within the course of an artery that either exceeded the diameter of the contralateral non-occluded vessel or that of the adjacent vessel segment on both the SWI and minimum-intensity-projection series [10] or diffusion restrictions on DWI distal to a continuous core DWI lesion. Peripheral emboli on DWI were related to findings on SWI and post-thrombectomy DSA. Peripheral emboli were classified as lesions within the vascular territory of the primary LVO or emboli into new territories.

All DWI lesions were segmented semi-automatically using seed growing algorithms provided by the Analyze 11.0 software package (AnalyzeDirect, Inc., Overland Park, KS, USA). Finally, probability maps were calculated.

DSA and MRI analysis were performed independently by two readers that did not participate in the endovascular therapy. Discrepancies between readers were resolved in consensus.

Data were reported using standard descriptive statistics. Spearman’s rank correlation test was used for correlations. Wilcoxon signed rank test was performed to compare continuous variables as appropriate. All statistics were calculated using SPSS 24.0 (IBM SPSS Statistics for Windows, Armonk, NY: IBM Corp.). *p* values < 0.05 were considered statistically significant.

## Results

Twenty-eight patients were included in the study. Prior to MT, alteplase was administered in 11 patients (39.2%). Details on baseline characteristics, procedural, and functional outcome of the patients can be found in Table 1.

**Table 1:** Baseline characteristics, procedural, and functional outcome

Baseline characteristics	All patients
	(*n* = 28)
Age in years, median (IQR)	71 (64.75–79.75)
Female sex, *n* (%)	13 (46.4)
Admission NIHSS, median (IQR)	14 (9.25–17)
Occlusion site, *n* (%)	
Terminal ICA	4 (14.3)
MCA	17 (60.7)
Tandem occlusion	7 (25.0)
ASPECTS, median (IQR)	7 (6–9)
Arterial fibrillation, *n* (%)	16 (57.1)
Procedural and functional outcome	
IV Alteplase, *n* (%)	11 (39.2)
Use of a distal aspiration catheter, *n* (%)	24 (85.7)
Use of BGC, *n* (%)	5 (17.6)
Primary aspiration/stent-retrieval with BGC/stent-retrieval with aspiration catheter	10/5/18
eTICI (2b50/2b67/2c/3)	4/9/6/9
Time symptom onset to recanalization in min, median (IQR) [*n* = 22]	260 (221.25–312.5)
mRS 90d, median (IQR) [*n* = 26]	2 (1–4)

*BGC* balloon guide catheter, *ASPECTS* Alberta stroke program early CT score, *ICA* internal carotid artery, *eTICI* expanded thrombolysis in cerebral infarction, *NIHSS* national institute of health stroke scale, *mRS* modified Rankin scale

**Fig. 1:**
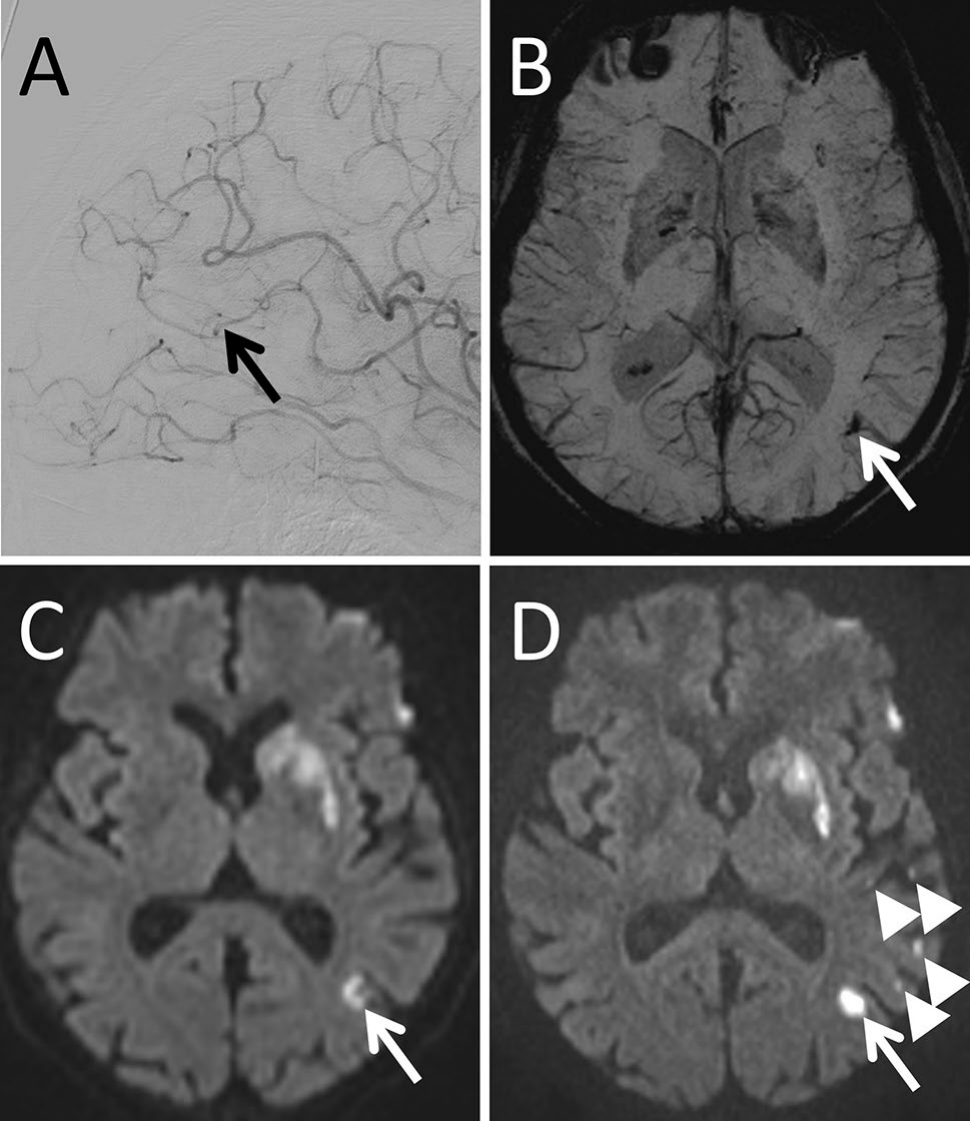
Example of a patient presenting with a left M1-segment occlusion that was successfully recanalized (eTICI 2b67). In correlation with an M3-segment occlusion on postinterventional DSA in lateral projection (**a**, arrow) a tubular signal drop can be seen in the follow-up SWI in minimal intensity projection (**b**, arrow). In the same location a diffusion restriction (arrow) can be found on regular DWI (**c**) and high resolution DWI (D). Additional small diffusion restrictions can be seen on high resolution DWI (arrowheads) that do not show a correlate in the DSA, SWI or standard resolution DWI

Twenty-six out of 28 patients showed peripheral punctuate lesions on high-resolution DWI suspicious for peripheral emboli. With a total of 324 lesions high resolution DWI detected significantly more peripheral emboli compared to standard resolution DWI (total 324 vs. 151; median 10 vs. 5; *p* < 0.001). 33 SWI lesions suspicious for peripheral emboli were found, all in close proximity to DWI lesions (33/324 = 10.2%). 8.0% (26/324) of DWI lesions were directly distal to occluded peripheral vessels on post-interventional DSA (Fig. 1). The majority of peripheral ischemic lesions was small with < 0.5 ml volume on DWI (online supplement Figure I).

The number of peripheral emboli was inversely correlated with eTICI scale (Spearman’s rho 0.611; *p* < 0.001). Eight (8/9) patients rated as eTICI 3 showed peripheral emboli. In 11 patients emboli into new territories were found in DWI (31/324 = 9.6%). The volume of peripheral lesions on high resolution DWI represented 2.1% of the cumulated ischemic stroke volume. The number of DWI and SWI lesions for each eTICI grade is shown in Table 2.

**Table 2:** Number of peripheral emboli on standard-resolution DWI, high-resolution DWI, and SWI for each eTICI grade and volume on high-resolution DWI

eTICI	2b50	2b67	2c	3
Number of patients	4	9	6	9
Number of patients without peripheral emboli	1	0	0	1
High-resolution DWI^a^	23.5 (5.5–29.5)	14.5 (9.75–28.75)	12 (8.75–14.25)	3 (1–4)
Standard-resolution DWI^a^	10 (0–12)	8 (4.75–11.5)	5.5 (4–7)	1 (0–2.5)
SWI^a^	1 (0–4.25)	0 (0–3)	1 (0–3.25)	0 (0–0)
Volume of peripheral DWI lesions [ml]^a^	1.0 (0.3–1.9)	1.3 (0.6–2.1)	1.1 (0.7–1.7)	0.1 (0.0–0.3)

*DWI* diffusion-weighted imaging, *SWI* susceptibility-weighted imaging, *eTICI* expanded thrombolysis in cerebral infarction

^a^Values are median number or volume of peripheral emboli (IQR)

There was a tendency towards a lower number of peripheral emboli when MT was performed under flow arrest using a balloon-guide catheter compared to MT with a median number of emboli of 5 (IQR 0.5–9.5) with and 11 (IQR 4–21.5) without a balloon-guide catheter but the difference was not statistically significant (*p* = 0.057) (online supplement Figure II). The number of emboli did not differ between patients that received IV alteplase and those who did not with a median of 11 (IQR 4–22) versus 17 (IQR 2.5–16) (*p* = 0.944).

**Fig. 2:**
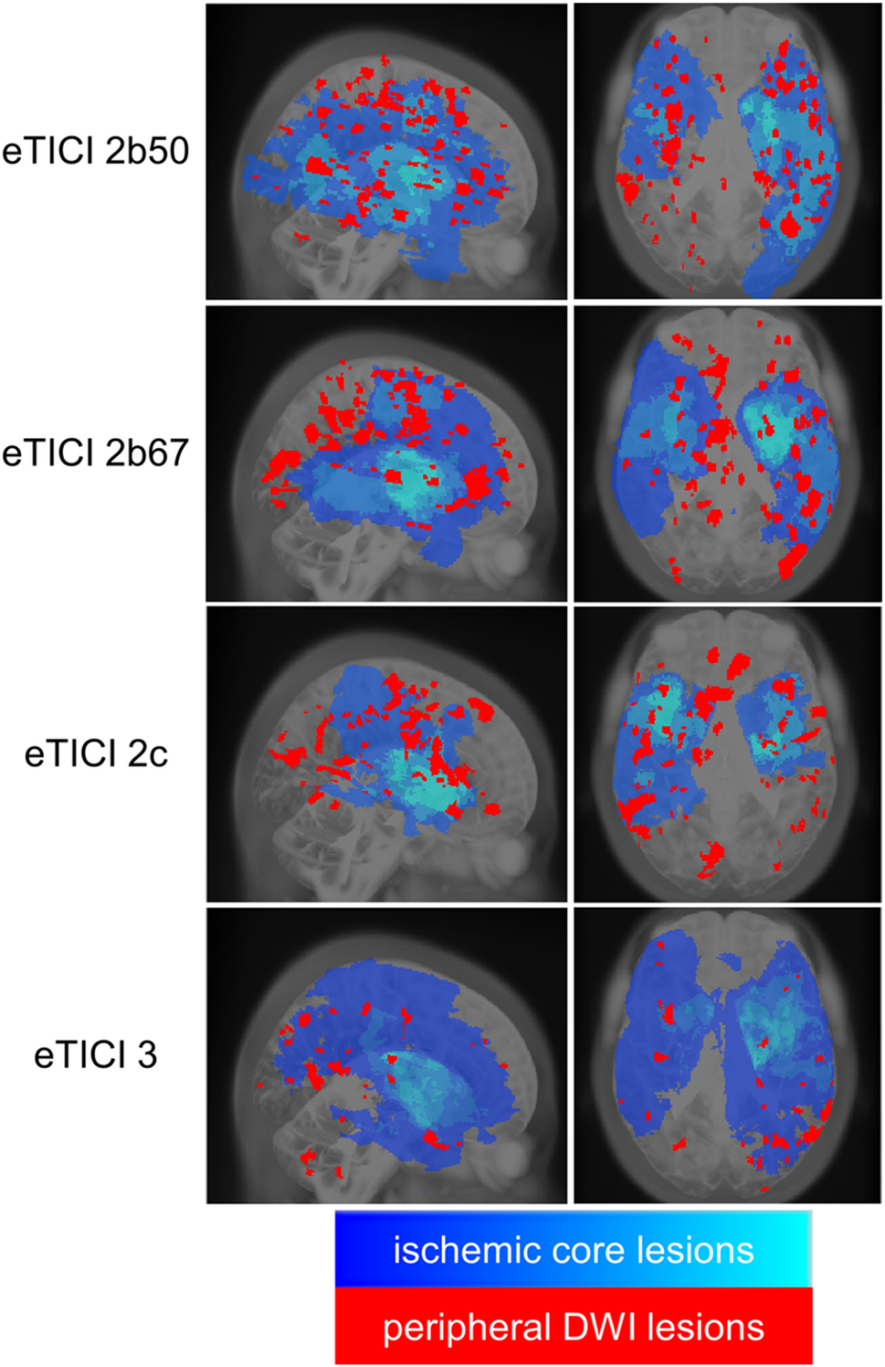
Probability maps of the ischemic core (high probability = light blue; low probability = dark blue) and peripheral DWI lesions (red) in sagittal (left) and axial (right) projection

Distribution of peripheral emboli in relation to the core DWI lesion for each eTICI grade is visualized in Fig. 2.

## Discussion

Our analysis confirmed the hypothesis that high-resolution DWI detects peripheral emboli within the vascular territory of LVO even after complete reperfusion (eTICI 3). Only a small fraction of DWI lesions occurred directly distal to DSA proven vessel occlusions. These sub-angiographic emboli occur much more frequently in the vascular territory of the initially occluded vessel than in other territories.

In vitro studies suggested that every endovascular recanalization attempt leads to peripheral emboli [14]. Previously, it was assumed that small peripheral emboli may dissolve spontaneously, thus, not leading to tissue infarction [15]. This is the first study to examine the degree of brain injury by peripheral emboli after MT using high resolution DWI. We observed that a certain amount of these emboli indeed cause peripheral tissue ischemia. Peripheral emboli were more frequent in eTICI 2b than 2c but occurred also in patients with eTICI 3.

Previous studies comparing pre- and post-interventional MRI have shown that over 90% of intracranial vessel occlusions are caused by a single thrombus [7]. In consequence, most incomplete reperfusions after MT (less than TICI 3) would result from thrombus fragmentation as a complication of the procedure. Reperfusion success was found to be the most important modifiable predictor for outcome in previous studies [5]. Consequently, persisting vessel occlusions after MT worsen clinical outcomes. However, the question why some patients with successful reperfusion and small core infarcts do not reach functional independency after 90 days remains incompletely answered. Hypertension, hyperglycemia, and fever are modifiable factors that are associated with worse outcomes after stroke [16]. Another factor might be peripheral emboli. In our study, total peripheral emboli volume represented only 2.2% of the cumulated ischemic core volume. Nevertheless, if located within strategic brain regions such as the motor cortex or cortico spinal tract, even small lesions might have a relevant effect on clinical outcome. Thus, it has been found that infarct location in addition to core infarct volumes is a significant factor determining the clinical outcome [17].

It was already shown that pre-interventional thrombus fragmentation worsens outcome in patients [7]. However, the relation of peri-interventional thrombus fragmentation to functional outcome has to be elucidated in further studies.

Our findings might have important implications for safety features during MT and design of future stroke studies. One focus of future studies will be the question if intravenous thrombolysis before mechanical thrombectomy is associated with an additional benefit [18]. If peri-procedural intravenous thrombolysis could reduce the burden of peripheral emboli as detected with DWI it might improve outcome. Currently, recommendations for procedural details are based on hypothetical considerations, in vitro testing and porcine in vivo models that are difficult to prove in the clinical setting [14, 19]. DWI infarct growth was proposed as a surrogate endpoint for future stroke therapy trials to reduce sample sizes [20]. Acquiring DWI in high resolution to detect peripheral emboli might further add to this metric.

It is possible that not all peripheral emboli may have been related to MT but could have occurred independently. But, as previously mentioned, over 90% of intracranial vessel occlusions are caused by a single thrombus [7]. Another argument for the association with MT is the clear inverse correlation of the number of emboli with the eTICI scale. Furthermore, a primary embolic source would have led to more emboli into new territories. Lastly, a peri-interventional occurrence of the emboli explains best the trend towards a lower number of emboli when using a balloon-guided catheter.

Another limitation is the limited sample size of the study. Future studies on larger samples using high-resolution DWI as follow-up will explore possible predictors for these emboli and their influence on clinical outcome.

## Conclusion

Emboli after MT for acute ischemic stroke occur most frequently within the vascular territory of an LVO, even after complete reperfusion of eTICI 3. High resolution DWI to detect peripheral emboli after MT could be a promising surrogate marker for reperfusion success beyond the TICI scale.

## Electronic supplementary material

Below is the link to the electronic supplementary material.

Supplementary file1 (PDF 42 kb)
