# Supplementary material for: Sub-angiographic peripheral emboli in high resolution DWI after endovascular recanalization
Source: J Neurol. 2020 Jan 29;267(5):1401–6. doi: 10.1007/s00415-020-09719-1 (PMC7184052; doi:10.1007/s00415-020-09719-1)
Supplement: Supplementary file 1 — Supplementary file1 (PDF 42 kb) [file 415_2020_9719_MOESM1_ESM.pdf]

SUPPLEMENTAL MATERIAL

Peripheral emboli in high resolution DWI after complete endovascular recanalization

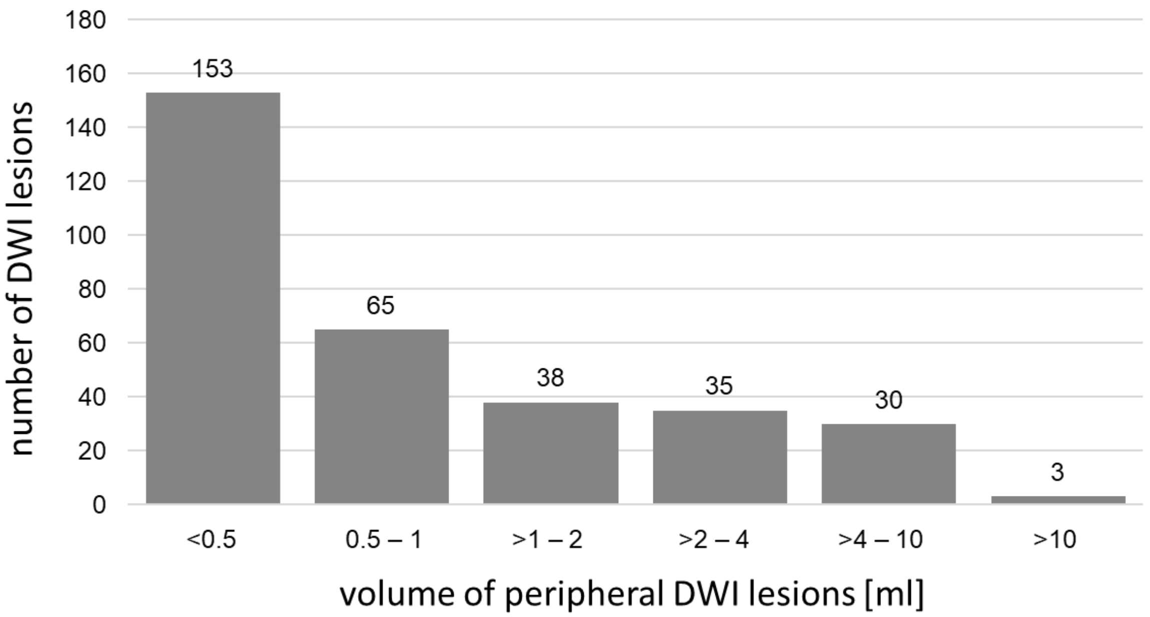

Figure I: Bar chart showing the distribution of peripheral DWI lesions sorted by their volume. 47.2% of peripheral lesions were smaller than 50ml.

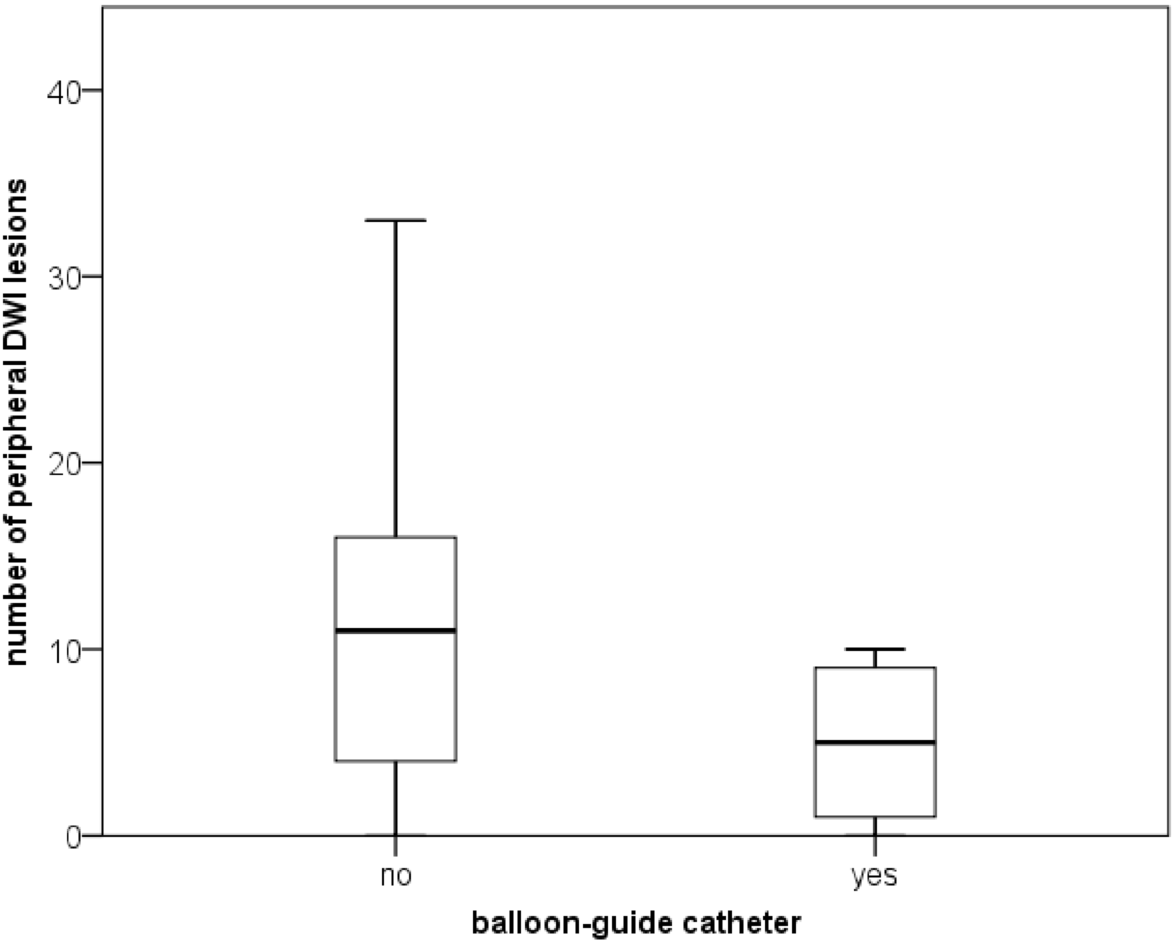

Figure II: Box plot showing a tendency towards a lower number of peripheral lesions on high resolution DWI with the use of a balloon-guide catheter (n=5; median 5, IQR 0.5-9.5) compared to not using a balloon-guided catheter (n=21; median 11, IQR 4-21.5). The difference was not statistically significant (p=0.057).
